# Book Review: The Role of Context in Language Teachers' Self Development and Motivation: Perspectives From Multilingual Settings

**DOI:** 10.3389/fpsyg.2021.724293

**Published:** 2021-07-13

**Authors:** Jieping Xu

**Affiliations:** College of Foreign Languages, Chaohu University, Hefei, China

**Keywords:** diverse sociopolitical contexts, individual differences, self-development, reflexive positioning, interactive positioning

Amy S. Thompson (Bristol: Multilingual Matters), 2021, xiii+163 pages, ISBN: 978-1-80041-117-3

It has been argued that research on teacher motivation and identity (re)construction requires the expansion of its traditionally psychological boundaries and embarks on the sociolinguistic and critical viewpoints borrowed from language learning, language teaching, and language teachers' lives in bilingual/multilingual environments. To cross these boundaries, Amy S. Thompson's monograph, entitled *The Role of Context in Language Teachers' Self Development and Motivation: Perspectives from Multilingual Settings*, is the result of 10 years of diligent work of collecting and analyzing the narrative data from English as foreign language (EFL) teachers in seven diverse sociopolitical contexts. The focus of the book is on individual differences, specifically motivation and the development of self, in bilingual/multilingual contexts.

This monograph includes a warm-in-tone forward, a preface, and nine chapters. Chapter 1 problematizes the complexities of integrating context, self-development, and motivation, by providing their germane theoretical underpinnings and a comprehensive overview of the pertinent issues such as non-native speaker status quo, formation of the idealized teacher identity and self, as well as context-specific teacher trajectories. Synthetizing the studies conducted on the interplay of context and self which were published in 17 journals from 2009 to 2020, the author cogently argues that there has been a dearth of empirical studies in some contexts which is the focus of this book. The chapter also reviews the empirical studies conducted in Africa, Southeast Asia, Latin America, the Middle East/Turkey, and Eastern Europe. Furthermore, the chapter elucidates the data analysis procedures, presentation of the narratives of NNS English language teachers in a variety of underrepresented contexts, as well as the methodology of collecting the narrative data from English as foreign language (EFL) instructors. Serving as a mentor, the author stated that the data were collected from a group of EFL teachers who attended an exchange program at the University of South Florida in the spring of 2010.The participants of this monograph were EFL teachers from different contexts, including Senegal, Egypt, Argentina Ukraine, Estonia, Vietnam, and Turkey.

Set in Senegal, in Chapter 2, Thompson reports that her EFL teacher conceptualizes the ideal English teacher self as someone who “believes that sharing a first language (L1) with students leads to an understanding of what they need, has a positive attitude toward English and speaks it whenever he can, and expresses that English is needed to succeed in various aspects of life, such as jobs, diplomacy, and exams” (p. 40). Chapter 3 focuses on the EFL teacher from Vietnam who opines that “the younger generation has more opportunities to learn English well and loves English after first conceptualizing it as a tool” (p. 51). To this EFL teacher, making an interpersonal relationship with students plays an important role and language learning is a “passport” to a brighter future, which culminates in a happier life.

Chapter 4 delineates the cognitions and perceptions that make the identity of an EFL teacher in the Egyptian context. This EFL teacher cogitates that “a person's education is often judged by their English level and that English is necessary in the globalized world” and “people do not ‘learn' a language but ‘live' a language” (p. 65). In Chapter 5, an EFL teacher from Argentina strongly believes that learning English “makes them empathetic to their students' processes” (p. 78) and that teachers need to feel accountable to foster teacher-student interpersonal relationships by creating positive attitudes and encouraging students. The context of Chapter 6 is Turkey where the two informant EFL teachers reflect that “English is a tool but can also create emotional attachments” (p. 90), and they believe that native English speaker is an advantage in hiring practices. The author recapitulates the attitudes of these two EFL teachers by stating that “Even though some people are opposed to the widespread use of English, most realize that it is currently a necessity for economic development on an international scale” (p. 102). In Chapter 7, two Ukrainian EFL instructors state that teacher-student interpersonal factors are vital to creating a positive and cheerful classroom atmosphere and that “English will open doors and is a mechanism for understanding people” (p. 103). Set in Estonia, the penultimate chapter explores how two EFL teachers feel about the role of motivation, context, and identity formation. They express positive attitudes toward English and argue that “sharing a first language (L1) with the students helps them know the specific difficulties that might arise” (p. 115). The closing chapter summarizes the sociopolitical, context-specific experiences of these EFL teachers from seven countries, argues that context is not a monolithic phenomenon, and elucidates how the construction of interpersonal relationships cannot be segregated from contexts. This chapter presents how the EFL teachers' images and visions of their professional and ideal selves have evolved in their sociopolitical contexts. The author concludes that “multilingual identity should be at the forefront of pedagogical discussions” (p. 135) and she believes that “emphasis should be placed on understanding the complex and dynamic relationships among language systems, as well as attitudes toward the different languages in a multilingual speaker's and/or teacher's repertoire” (p. 135).

I believe that this monograph is thought-provoking in that it delves deeply into the professional and academic trajectories of EFL teachers who come from diverse sociopolitical, emotional, cognitive, and behavioral backgrounds. Furthermore, the qualitative data were collected from teachers from seven countries which were underappreciated in the literature, so the findings can be novel and insightful. If the author had taken into account reflexive positioning—individuals' positioning of themselves—and interactive positioning—individuals' positioning of others into account (Kayi-Aydar, 2018), the findings would have been more insightful. All in all, I believe that this book is an appealing read for teachers, students, teacher educators, and policymakers in EFL contexts.

## Author Contributions

The author confirms being the sole contributor of this work and has approved it for publication.

## Conflict of Interest

The author declares that the research was conducted in the absence of any commercial or financial relationships that could be construed as a potential conflict of interest.
